# Effect of endodontic treatment on periodontal healing in Grade III EPL without root damage

**DOI:** 10.6026/973206300223328

**Published:** 2026-06-30

**Authors:** Meetu Mathur, Shreyansh Ahirwar, Dhanuja Rani J., Srinivas Murthy S.T, Aseem Jolly Garg, Micky Kumari, Miral Mehta

**Affiliations:** 1 Department of Conservative Dentistry and Endodontics, Rajasthan Dental College and Hospital, Nirwan University, Jaipur, Rajasthan, India; 2 Department of Dentistry, Bundelkhand Medical College and Hospital, Sagar, Madhya Pradesh, India; 3 Department of Oral and Maxillofacial Pathology, Government Dental College and Research Institute, Ballari, Karnataka, India; 4 Department of Pedodontics and Preventive Dentistry, Government Dental College and Research Institute, Ballari, Karnataka, India; 5 Department of Conservative Dentistry and Endodontics, Tantia University, Sri Ganganagar, Rajasthan, India; 6 Department of Periodontics, Hi Tech Dental College and Hospital, Bhubaneswar, Odisha, India; 7 Department of Pedodontics and Preventive Dentistry, Karnavati School of Dentistry, Karnavati University, Gandhinagar, Gujarat, India

**Keywords:** Clinical attachment level, endo-periodontal lesions, grade 3 endo-periodontal lesions (EPL), periodontal healing, probing depth, root canal therapy, tooth mobility

## Abstract

Periodontal and endodontic infection, with or without root damage, go in conjunction with destruction of periodontal bone structure. Therefore, it is of interest to evaluate the effect of non-surgical endodontic treatment of periodontal healing in Grade 3 endo-periodontal lesions (EPL) without root damage. Hence, a total of 100 patients received standardized root canal therapy and were followed for six months. Significant improvement was observed in probing depth, clinical attachment level and tooth mobility. Thus, we show endodontic treatment as an effective approach for managing such lesions conservatively.

## Background:

Endo-periodontal lesions (EPLs) represent a complex pathological condition characterized by the interplay between pulpal and periodontal disease pathways, often leading to severe tissue destruction if inadequately treated [1, 5]. The 2018 classification of periodontal diseases by the European Federation of Periodontology and the American Academy of Periodontology redefined EPLs as conditions associated with periodontal and endodontic infection with or without root damage [2, 6]. Among these, Grade 3 EPLs without root damage are particularly challenging, as they exhibit deep periodontal pockets and radiographic bone loss, often mimicking true periodontitis, yet may originate from endodontic infection [3, 7]. In the past, treating EPLs usually involved both root canal therapy and periodontal treatment. However, more recent evidence shows that primary endodontic infections can cause periodontal symptoms that may improve with endodontic therapy alone, especially when there are no root fractures or perforations [5, 8]. Early studies by Simon *et al.* and others found that pulpal disease can spread to the periodontal ligament through structures like lateral canals and dentinal tubules [5]. Recent studies have substantiated this link with clinical data showing periodontal healing following endodontic treatment, even in advanced lesions. For instance, Ballal *et al.* (2021) demonstrated radiographic and clinical resolution of EPLs after RCT in a majority of cases, without the need for surgical periodontal intervention in the short term [1]. Similarly, Rossi-Fedele *et al.* (2023) and Chen *et al.* (2024) reported significant reductions in probing depth (PD), clinical attachment level (CAL) and tooth mobility in patients with Grade 3 EPLs treated with endodontics alone [3, 4]. Herrera and colleagues, through systematic reviews and consensus reports, stressed that proper diagnosis and starting treatment with endodontic therapy in true endo-perio lesions is key for good results [2, 6 and 7]. Their work showed that while some cases may later need periodontal therapy, beginning with endodontic treatment often lets inflammation resolve naturally and can prevent unnecessary procedures [6, 7]. Wang *et al.* highlighted that controlling infection is crucial for successful root canal treatment. They found that proper cleaning, filling and sealing of the canal are important for healing both around the root tip and in the gums [8]. Tietmann *et al.* (2023) an other such studies supported this by showing that patients with Grade 3 EPLs treated with root canal therapy had stable teeth and a survival rate of over 90% after seven years [9, 10, 11]. This study adds to existing research by looking at clinical and X-ray results over 12 months after endodontic treatment alone in Grade 3 EPL cases without root damage. Out of 100 participants, 92 completed the follow-up. The results showed significant improvements in probing depth, clinical attachment level, bleeding on probing and mobility. Periapical healing was seen in 86% of cases after 12 months. These results support the idea that controlling infection with root canal therapy alone can help periodontal healing in certain EPL cases. Gupta *et al.* and Schmidt *et al.* and other such studies have suggested that the timing and order of treatments may not be as important as once believed, as long as the endodontic infection is properly treated [10, 12 and 13]. This supports a conservative approach are start with endodontic therapy, monitor the patient and only consider periodontal surgery if the problem does not improve [14, 15]. Therefore, it is of interest to find out if endodontic treatment alone can lead to periodontal healing in Grade 3 EPLs without root damage and how much clinical measures improve over time.

## Methodology:

This prospective observational study looked at how endodontic treatment affects periodontal healing in Grade 3 EPL without root damage. One hundred patients aged 25 to 60 were selected based on clinical and X-ray diagnosis of primary endodontic lesions with secondary periodontal involvement and no root fractures, perforations, or resorption. Eligible patients were generally healthy, did not smoke or smoked only moderately and had not recently received periodontal therapy or medications that could affect healing. People with advanced tooth mobility, root damage, or systemic diseases were excluded. Baseline measurements included PD, CAL, BOP, mobility and either CBCT scans or periapical X-rays. All patients received the same endodontic treatment, which involved preparing the access cavity, cleaning the canals with rotary NiTi files, irrigating with sodium hypochlorite and ethylenediaminetetraacetic acid (EDTA), placing calcium hydroxide (Ca(OH)2) and filling the canals using the lateral condensation method. One operator performed all treatments. No periodontal therapy was given during the first three months to focus on the effects of endodontic treatment. Follow-up checks for PD, CAL, BOP, tooth mobility and bone healing on X-rays were done at 1, 3, 6 and 12 months. The same examiners, who were trained for consistency, did all the clinical and radiographic assessments. Intra-examiner reliability was measured using intraclass correlation coefficients. Statistical analysis was done with SPSS v26.0, using a significance level of P < 0.05 and either paired t-tests or Wilcoxon signed-rank tests as needed.

## Results:

Ninety-two of the 100 participants who were enrolled finished the 12-month follow-up. After only endodontic treatment, without any additional periodontal therapy during the first three months, the clinical and radiographic parameters demonstrated a statistically significant improvement. Out of 100 enrolled participants, 92 completed the 12-month follow-up. During the first three months, only endodontic treatment was given and both clinical and radiographic measures showed significant improvement. After 12 months, the average PD fell from 7.2 mm to 3.8 mm (P < 0.001). CAL improved from 7.8 mm to 4.0 mm (P < 0.001). BOP dropped from 89% to 18%. The number of teeth with moderate to severe mobility decreased from 65% to 12%. Radiographs showed that 86% of treated teeth had healed periapically. These results indicate a significant reduction in attachment loss and periodontal inflammation after root canal therapy. Most improvements happened in the first six months, with healing continuing more slowly but steadily after that. These findings support the idea that in Grade 3 EPL cases without root damage, controlling infection in the root canal alone can lead to periodontal healing. Table 1 gives a detailed summary of clinical parameters over time.

## Discussion:

This study shows that for Grade 3 EPL without root damage, nonsurgical root canal therapy alone can lead to significant periodontal healing. Improvements in PD, CAL, BOP and tooth mobility support the endo-first treatment approach. These results fit with current knowledge about the connections between pulpal and periodontal tissues. The development of EPL, especially when there is no root damage, is linked to anatomical pathways like accessory canals and apical foramina that connect the pulp and periodontium. López Marcos (2004) noted that these channels let toxins and bacteria move from the root canal to the surrounding tissue, making it important to remove pulpal infection first [16]. In these cases, controlling the main infection with proper endodontic cleaning and sealing can reduce or stop secondary periodontal inflammation. A 2025 case report in Cureus also supported this approach, using a multidisciplinary protocol for Grade 3 EPL without root damage. The treatment included step-by-step root canal therapy, careful coronal sealing and delayed non-surgical periodontal therapy. After three years, the patient showed stable periodontal and periapical health [17]. These results support the protocol in this study, where periodontal therapy was delayed for three months to focus on endodontic healing. Fan *et al.* (2020) also found that root integrity, no vertical fractures and good canal disinfection predicted better results when using root canal therapy alone or with scaling and root planing for Grades 2 and 3 EPL [18]. Tietmann *et al.* (2021) highlighted that the shape of the defect and use of regenerative materials affect healing. Their analysis showed better outcomes in teeth with narrow or shallow defects, similar to those in our study group [9]. A systematic review in 2023 found that root canal therapy alone in Grade 3 EPL without root damage led to an average PD reduction of 3.2 mm, a clinical attachment gain of 2.3 mm and a 45% radiographic healing rate at 12 months [3]. In our study, these improvements were even greater, likely because of strict selection criteria, consistent treatment and careful coronal restorations, which help periapical healing. An expert consensus in the International Journal of Oral Science (2024) also stressed the value of the endo-first approach. They advised waiting to start periodontal treatment until after root canal therapy, unless symptoms lasted longer than expected [19]. This step-by-step method matches the evidence-based prognosis model by McGowan *et al.* (2017), which considers lesion origin, probing depth and root surface condition in making treatment decisions [20]. Tsesis *et al.* (2019), in their textbook on endodontic-periodontal lesions, described clinical protocols that put root canal therapy first when treating combined lesions. They recommend monitoring how teeth respond to endodontic therapy before moving to surgical or regenerative periodontal treatments. This approach matches the framework of our study [21]. The model is based on early work by Simon *et al.* (1972), who first classified EPL and identified those that could heal with root canal therapy alone [5]. A study by Ruetters *et al.* concluded that endodontic therapy alone resulted in treatment success for some of the teeth, which would otherwise have had a poor prognosis [22]. In summary, this study confirms that for carefully chosen Grade 3 EPL without root damage, nonsurgical root canal treatment alone can lead to significant periodontal and periapical healing. These results are strongly supported by anatomical, clinical and evidence-based research. A systematic, step-by-step approach that starts with endodontic treatment then evaluates and adds periodontal therapy if needed, gives the best outcomes for these cases.

## Conclusion:

For Grade 3 EPL without root damage, endodontic treatment alone can greatly improve periodontal healing. Good results depend on through root canal cleaning and choosing the right cases. In some situations, remaining pockets may need extra periodontal therapy.

## Advancement to knowledge:

Grade 3 EPLs without root damage, nonsurgical root canal treatment single handedly can lead to significant periodontal and periapical healing. Hence the clinical efficiency and patient satisfaction is significantly improved.

## Figures and Tables

**Table 1:** Clinical parameters at baseline, 3, 6 and12 Months (Mean ± SD)

**Parameter**	**Baseline**	**3 Months**	**6 Months**	**12 Months**	**P-value (Baseline vs 12 Months)**
Probing Depth (PD, mm)	7.2± 1.1	5.1± 1.0	4.3± 0.9	3.8± 0.8	<0.001
Clinical Attachment Level (CAL, mm)	7.8± 1.2	5.5± 1.0	4.7± 0.9	4.0± 0.8	<0.001
Bleeding on Probing (BOP, %)	89%	52%	31%	18%	<0.001
Tooth Mobility (Miller Grade ≥ II)	65%	39%	22%	12%	<0.001
Radiographic Periapical Healing (%)	-	58%	72%	86%	-
